# Between a woman and her fetus: Bedouin women mediators advance the health of pregnant women and babies in their society

**DOI:** 10.1186/s12884-021-03661-4

**Published:** 2021-03-06

**Authors:** Rachel Sharaby, Hagit Peres

**Affiliations:** Ashkelon Academic College, Ashkelon, Israel

**Keywords:** Reproductive/perinatal health promotion, Interpretive feminist inquiry, Narrative analysis, Ethics of care

## Abstract

**Introduction:**

Bedouin women in Israel confront a challenging circumstance between their traditional patriarchal society and transition to modernity. In terms of reproductive health, they face grave disparities as women, pregnant women and mothers. In this article we aim to understand the challenges of Bedouin women who work as mediators in the promotion of Bedouin women’s perinatal health. We explore their challenges with the dual and often conflictual role as health peer-instructors-mediators in mother-and-child clinics, and also as members of a Bedouin community, embodying a status as women, mothers, and family caretakers. Drawn upon a feminist interpretative framework, the article describes their challenges in matters of perinatal health. Our research question is: how do women who traditionally suffer from blatant gender inequality utilize health-promotion work to navigate and empower themselves and other Bedouin women.

**Methods:**

Based on an interpretive feminist framework, we performed narrative analysis on eleven in-depth interviews with health mediators who worked in a project in the Negev area of Israel. The article qualitatively analyses the ways in which Bedouin women mediators narrate their challenging situations.

**Results:**

This article shows how difficult health mediators’ task may be for women with restricted education who struggle for autonomy and better social and maternal status. Through their praxis, women mediators develop a critical perspective without risking their commitments as women who are committed to their work as well as their society, communities, and families. These health mediators navigate their ways between the demands of their employer (the Israeli national mother and child health services) and their patriarchal Bedouin society. While avoiding open conflictual confrontations with both hegemonic powers, they also develop self-confidence and a critical and active approach.

**Conclusions:**

The article shows the ways by which the mediator’s activity involved in perinatal health-promotion may utilize modern perinatal medical knowledge to increase women’s awareness and autonomy over their pregnant bodies and their role as caregivers. We hope our results will be applicable for other women as well, especially for women who belong to other traditional and patriarchal societies.

**Supplementary Information:**

The online version contains supplementary material available at 10.1186/s12884-021-03661-4.

## Introduction

This research describes the complex ways in which small group of Bedouin women who were recruited and trained as peer-instructors and mediators for promotion of the health of Bedouin women of fertility stage. They encounter numerous challenges as they navigate between their own Bedouin society with its cultural conventions and patriarchal characteristics, and the medical service that employs them. The purpose of this research is to understand the social mechanisms that stimulate social equality and change, and to understand social and cultural costs paid by women promoters of change and their efforts to avoid open conflictual confrontations with the traditional patriarchal forces in the Arab-Bedouin society in the south of Israel.

Bedouin society is originally a traditional-nomadic society, a minority within Muslim society in Israel, and in spite of the changes taking place in it, the patriarchal structure is dominant and central and is zealously preserved [1–4]. In 2020, Bedouin society in the south of Israel included approximately 270,000 people, comprising around 25% of the Negev population [5–7]. However, until the establishment of Israel in 1948, the Bedouin in the south of Israel subsisted mainly on raising goats and small farming. Upon the establishment of the state, the Bedouin population was obligated to undergo dramatic changes in its lifestyle, which included a disintegration of the original tribal structure and undermining of their traditional economy and lifestyle in favor of a market economy, and a sedentary life and modernization [8, 9].

The State of Israel was unsuccessful in accompanying these rapid transitions and in supplying the necessary socioeconomical support and resources for such a drastic social change. Currently, approximately 90% of the Bedouins in the Negev belong to the lowest percentile [9, 10]. Most of the older men have difficulties in becoming included in the modern market economy and find their livelihood in menial labor or unemployment. Most of the older women remained restricted and dependent on the men both socially and financially. The health and education of Bedouin women and their children is therefore poor, compared to that of the Jewish population in the south of the country [4, 11–17].

Some women of the younger generation are finding the strength to become included socially and economically by acquiring an education and economic independence. More and more women drive a car, acquire an education or professional training, and opportunities to earn a living independently [12, 16, 17]. Nonetheless, the gap between the small number of independent and empowered women and most women who still live within the restrictions of traditional Bedouin life is immense [17].

### Feminists’ criticism of the patriarchal perspective of care, childbearing and caretaking for the other(s)

Many women around the world suffer from gender inequality. Rigid patriarchal structures provide men normative access and control over resources, power and authority from which women are excluded. De Beauvoir [18] was a pioneer in expressing public criticism of the prevalent belief in the biological superiority of men, and against the discursive legitimization of women as weak and passive biological entities, suitable solely for giving birth to offspring, caring for them and managing the household. Ortner [19] continued by defying the social perception of women in a category that is closer to nature than to culture, and thus confines women to the boundaries of the domestic sphere [20]. Numerous feminist researchers from the mid-twentieth century strongly opposed the connection of femininity confinement to domesticity, directing its formulation by patriarchal hegemony [21, 22]. Several scholars suggested that feminine inferiority expressed in the exclusion of women from participation in the public sphere was intended to fulfill the economic and political interests of the masculine hegemony [23–26].

Feminist criticism has become even more scathing with reference to the patriarchal discourse which turns women’s reproductive abilities into a weakness and lack of power, instead of recognizing women's force that creates life, nourishes, and raises the human beings. Sarah Ruddick [27] wrote in her book *Maternal Thinking*:

“In most societies however, women are socially powerless in respect to the very reproductive capacities that might make them powerful. The primary bodily experience of mothers is a poignant reminder that to think of maternal power is immediately to recall maternal powerlessness – and conversely. …. Children confront and rely upon a powerful maternal presence only to watch her become the powerless woman in front of the father, the teacher, the doctor, the judge, the landlord – the world.”

In recent decades, feminist researchers have challenged the thinking about care and caring as an inferior and excluding attribute that characterizes women, arguing for women’s affinity for care as qualities worthy of social recognition and esteem, and for suitable economic compensation.

Fisher and Tronto [28] were the first to define *care* in a gender-feminist context, as follows: “Care is a species activity that includes everything that we do to maintain, continue, and repair our ‘world’ so that we can live it as well as possible. That world includes our bodies, ourselves and our environment, all of which we seek to interweave in a complex life-sustaining web” (p. 40). The paradigmatic change led by current feminists views of the domestic sphere as a space that enables women to develop a perception of responsibility, sensitivity and caring for others and their needs, without negating their capability to care for their personal growth also within the framework of the public sphere. Thus, care and caring do not need to comprise a barrier for women’s advancement and prevent them from obtaining equal rights, social esteem, and social privileges [29–34].

### Women in the patriarchy of the traditional Bedouin society

Bedouin tradition demands modest behavior from women. Traditional dressing must cover most of their body (the rigorous ones also cover their face, leaving only an eye slit). Marriages are usually arranged by parents, with a clear preference for cross-cousin marriage within the patrilineal and patrilocal family. Most of the young couples live near the husband’s family. Young brides are compelled to leave their original families to live in the husbands’ patriarchal households [15].

Bedouin women are expected to be prolific housewives and mothers. Alhuzeel-AlAssad (2013) presented the traditional restraining of women in Bedouin society as a conditioned act in an unwritten inter-gender pact: as long as she maintains the rules of obedience to men and the behavior codes expected from her, including avoiding appearance in the public domain, her honor, body and livelihood should be ensured [3].

Judicially, Israeli Bedouin women are acknowledged with equal rights as any other Israeli citizen, including rights to consume health, welfare, and legal services. However, the acquisition of egalitarian rights for most Bedouin women is noticeably slow, compared to their surroundings. With time and modernization, the Bedouin “classical patriarchal contract” that confines the role of women to giving birth and caring for the children and the house, and the traditional entitlement of men as responsible for supervising their women, are undergoing gradual changes due to the necessity of including women in the family’s livelihood [1, 2, 14, 15].

However, the feminist project that aspires to draw women into the modern world often ignores such traditional cultural and gender codes, and that may turn the women into victims of modernization [17]. There is a current thinking in the feminist literature that any social change that draws minority societies to Western culture is a beneficial change. Two Bedouin women researchers, Nuzha Alhuzeel-Alassad [3] and Sarab Abu-Rabia-Queder [12, 15], criticized this common conception by claiming that change and drawing near to the Western world are not always beneficial for women who belong to traditional groups. Harel-Shalev et al. (2018) [1] added and described a complex reality where modernization does indeed offer opportunities for many young Bedouin women to acquire education and means for mobility in the public sphere as well as to earn independency. However, the masculine control does not disappear, and is sometimes felt even stronger and in numerous symbolic ways. Harel-Shalev et al.’s study [1] showed that for many Bedouin women, the urbanization process even blocked possibilities of spatial movement within the new public space of clinics of welfare and health services. Thus, these women continue to negotiate and struggle with the masculine aim to limit their actions. Those women who burst the barriers of tradition in favor of livelihood and public activity cope with serious and sometimes impossible pressure [17].

### Women’s reproductive health and wellbeing under rigorous patriarchal societies

The field of health demonstrates the extent to which patriarchal social mechanisms pose difficulties for women, particularly women from weakened groups, in the maintenance of their health and the health of their children [35]. Marmot’s pioneering work on the social determinants of health indicated gender ascription as a major health determinant [36]. However, poor health is not an independent factor. Rather, it is a product of social contexts that impart women less power and resources for realizing good health for themselves and their children [37–39]. Many women are under patriarchal supervision that limits their freedom and mobility in space and their independence. In absolute terms, children of women who did not have an opportunity to acquire an education may have a 50-fold greater risk of suffering from violence, malnutrition and/or infant mortality until age five [40, 41].

In many traditional societies, women’s health is influenced by numerous factors: close social control over their sexuality and reproductive abilities; masculine hegemonic control that is supported in many cases by religious laws and includes marriage arrangements; prohibition and severe punishment for extramarital sexual relations; restriction on utilization of medical control of birth and family planning by supervision over access and selective use of health services and medical technologies, including use of contraceptives, as well as limiting the use of prenatal tests and pregnancy terminations. Women in societies controlled by the masculine hegemony are thus perceived as being able to bring children into the world, but not as being able to make decisions pertaining to their and their babies’ bodies, through reproduction and birth processes [2, 42].

Furthermore, restriction of access to family planning and expectation for multiparity and raising large families imposes frequent pregnancies and care of multiple offspring that exacerbate health risks for the women and their children, and increase the women’s dependence on men for their livelihood. Studies show a high prevalence of bad birth outcomes among marginal minorities, including low birth weight, premature births, and infant mortality [11, 40, 41, 43]. This creates a vicious circle as women who are prevented from controlling their health and status, give birth to more vulnerable children, and this exacerbates families’ vulnerability and dependence on a masculine support that is not always able to supply the needs of their weakened women and children.

Mindful of these observations, we studied a small and determined group of Bedouin women as they navigate between their traditional patriarchal communities and a demanding health service that employed them as peer-instructors and mediators to deliver reproductive health knowledge in mother-and-child (MCH) clinics. We examined how these women health workers, who traditionally suffer from blatant gender inequality, utilize health-promotion work to navigate and empower themselves and other Bedouin women. We also intended to learn what best social support could an employer, such as a health provider, offer its employees as they struggle to perform their roles as peer-instructors and intercultural mediators in the health promotion context.

## Methods

### Context

Infant mortality rates in the Bedouin society in Israel are significantly and consistently higher compared to the general population [44, 45]. Traditionally, many Bedouin women still marry men within their extended family (with preference for paternal cousins) [2, 45–47]. This custom of consanguineous marriage increases the risk for genetic defects, which is a dominant factor in the infant mortality rates in Bedouin society [44, 46, 47]. Other related factor for increases in infant mortality rates are universal consequences of difficulties faced by marginalized women living in weakened and poor environments facing scarcity and restrictions in mobilizing resources, and failing to control their own and their children’s health [48–50]. Similar difficulties are faced by Bedouin families when failing to provide basic necessities due to poor living conditions [2, 11].

This was the motive for establishment of the “Program for Reducing Infant Mortality and Health Disparities in Bedouin Society” which was implemented for two decades, between 1995 and 2015, by the Ministry of Health (MOH) and the Faculty of Health Sciences (FOH) at Ben Gurion University in the Negev with collaboration of other relevant health services.

The women who were recruited to work as health mediators had secondary education and were given an exceptional employment opportunity to be health instructors-mediators. As it will be shown in this article, they were trained to disseminate medical perinatal information elaborated by health authorities, and concomitantly experienced unique challenges as women aspiring for a change in their own traditional and patriarchal society. At any given time, eight to fifteen mediators were employed and trained in the project. They worked at MCH clinics that serve the Bedouin society in the south of Israel.

The main role of the Bedouin mediators, who speak the local Arabic dialect, was “Bridging over language and culture barriers between the Hebrew-speaking treating medical team and the Arabic-speaking Bedouin women who visited to the clinic” (cited from the projects’ goal). The project’s objectives included: raising the level of knowledge and awareness regarding risks for the fetus during pregnancy and baby; recommended behavior for reducing the risk of birth of congenital defects; and improving the utilization of health services for women of childbearing age and babies, education for the improvement of women’s nutrition during pregnancy, avoiding domestic casualties, and more. As will be shown below, the mediators’ work actually turned out to be much more complex than initially planned.

One of the authors participated in the program’s operation for almost a decade, and her role included recruitment of health mediators, training them during the course of their work, writing culturally adapted instructive materials and performing a qualitative evaluation for the project. The qualitative in-depth interviews were performed at the final stage of the project in 2015.

### The participants’ characteristics

The mediators were chosen for the role based on job interviews, participation in several weeks of intensive preliminary training and rending a summarizing exam at the end of training. Acceptance criteria included 12 years of education, good mastery of Hebrew and good human relations. High preference was given to women-mediators who were themselves mothers. Table 1 (see Appendix) presents characteristics of the interviewees at the time of the interviews in 2014. We used initial letters instead of full names, mean ages, years of education and mean number of children to maintain their confidentiality.

### Data collection

With the aim of obtaining the told narratives of the women mediators, we approached each one of them and asked their assents to be interviewed in an in-depth unstructured mode. This kind of interviews is the most suitable for obtaining authentic narratives [51, 52]. The face-to-face encounter with the interviewed women strives to contribute to the establishment of trust relations between investigators and participants. The encounter enables confirmation of meanings, and thus maintains the credibility of the investigators’ interpretation. This is particularly important when the investigator and the interviewed women come from different cultures [53]. Furthermore, collecting the life stories of the “others”, the “marginal”, and placing them at the center of the discussion, exposes not only their world, but also enables the researcher to explore the findings within a broader theoretical context [54].

The eleven interviewed women-mediators who were well acquainted with one of the authors voluntarily agreed to be individually and confidentially interviewed. Each one chose the time and place for her interview (MCH clinic, the project’s office, a café or her own house). The interviews were conducted in Hebrew and they flowed freely and vividly in a very confident atmosphere. All interviewees received an introduction on the objectives of the interviews and provided their informed consent to the use and publication of the collected materials. To avoid formality, interfere the trust and confident atmosphere in the interview, their informed consent was audio-recorded along with the interviews. The audio records were transcribed and analyzed. The interview guide translated to English is enclosed in this paper as Additional file 1.

### Data analysis

One of the main qualities of the narrative approach is the perception that people are natural story tellers, and that stories express the meanings that compose important aspects of the narrator’s own cultural background. Narrative analysis emphasizes the communicative elements of stories and enables participants to choose and express life experiences through their own cultural perspectives. The story thus has an organizing power [55–57]. As researchers we learn from narrated stories by interpreting the points of view of the story tellers’ viewpoints regarding the studied phenomenon. We approached the data analysis by focusing on the content (themes and categories) as well as on the structure of the narrated story. We also paid close attention to the diverse contextual backgrounds (time and place) chosen by the interviewees and what they contributed to the “stories”. The narrative approach is common in feminist research as it focuses on excluded marginal groups whose narratives as a group or as individuals are not afforded social, cultural, or political expression [57–59]. By locating and listening to these silenced voices, it is possible to learn about cultural contents that guide women and the historical and social contexts that influenced the consolidation of their identity [60–62]. Special focus was given to both spoken and subtle expressions of criticism as well as to the social position of the narrator and what this can tell about social forces taking place in each narrated scenario. The present study was carried out with a focus on the social relations of the health mediators, particularly on the way the women experienced themselves and the power relations of the patriarchal structure [56, 57]. In feminist thinking, emphasis is placed on the relation between feminine empowerment and knowledge, i.e. education and learning, as generating change [58]. Our attention was drawn to the creation of a new epistemological agenda, in which researchers pointed to the way science reflects only men’s fields of interest and excludes their experiences as women who hold their own topics of interest and perspectives [56, 63].

As women from the Bedouin society, our participant-informants were employed and trained to communicate medical messages to promote health and reduce the recurrence of perinatal health problems among Bedouin women living in Israel. They proudly shared their stories with us, aiming to illuminate their challenges as women who traditionally suffer from blatant gender inequality in their determined struggle to take advantage of the opportunity to promote social change for themselves and for other Bedouin women.

## Results

The mediators worked in MCH clinics, during the morning hours, which is convenient for mothers. However, great effort is required of mothers who live in unrecognized villages to reach their place of work on time. Furthermore, the families restrict them to work only in certain clinics, and often forbid them to continue with their role either temporarily or permanently. They received a low monetary compensation for their work, and sometimes, when the project’s budget ran low, their working hours were cut. As mentioned, this work was a rare opportunity for them to find employment, especially employment that was intellectually and socially stimulating.

### To help women put their health in the center

Motherhood is a central and determining experience in the traditional lives of Bedouin women, and affords them social status. However, together with other MCH women-patients, they experience huge resentment around their low position in the social hierarchy, placing themselves and their health as mothers in last place, and often even neglecting their own health issues. Eventually, self-neglected health may also harm the babies’ health.

**M.**: *I see the woman’s face and recognize what she needs. I explain to her what needs to be done, very slowly, I tell her: I am here for you. If you want a healthy baby, then take care and do something good for yourself, this is not a joke. The nurse says that her hemoglobin is very low, and she (already) has four children. It always pains me to see the woman walk like that. You see in her face that she is always tired. And I tell her about the tests and ask, why do you have a child every year? And why don’t you take care of your health?*

**I.:** *As a mother, I feel that this baby is the woman’s entire life. I am sure that every mother has this feeling. The baby is her entire world, something important that I feel good about. Health for the Bedouin women is on the last floor. I can ruin my life just so that my husband will be happy. How is my health? I do not care. The most important thing for me is that my husband and children will be healthy, and I do not care about myself. I will get up and do whatever is necessary. We have one patient who hemorrhaged every six months, and I told her to go to the doctor for a checkup. She has older children at home, but she said, who will stay with my [young] children? She does not care about herself. Her baby’s health is important for her, but not her own. Her life is over. It no longer has any value.*

### “I Am One of Them”: providing peer-group support through mediation

As Bedouin women who share daily complex relations within their communities, the mediators are required to tread carefully and out of awareness to womens' aprehension and resistance that may arise. During the workshops that were held in the project for advancing the health of pregnant women and babies, and particularly in role-acting games, the mediators shared their frustrations when expressions of resistance by peer women who rejected some of the messages communicated during instruction encounters in the clinics. The mediators shared the difficulty of confronting oppositions from women who publicly expressed indignation, anger and even derision of the messages and the modern medical perinatal treatment. The instructed women challenged the mediators publicly regarding their loyalty to Bedouin traditional and religious conventions, challenging the mediator’s loyalty to their own society. These confrontations compelled the mediators to develop awareness, skills and methods for coping with such challenging situations. Some common communicative skills and messages developed by the mediators are presented below:

**N.:** *I am one of them, and I did not like to be treated [otherwise]. In social terms this is fine. The people are happy to recognize and speak and share. To ask for my help. People are not afraid and do not keep their distance from me.*

**B:** *I am not a nurse or a doctor, I am a human being. Sometimes I tell the women that yes, you also have knowledge, and I also learn from you. It is not as if I am the boss here … And I speak to them at their level. They need this. When you speak to a woman from above, from up high, she will not accept you. You must know how to speak with them. Yes, we are both humans. I am not better. I also do not know everything, and there are things that perhaps she knows, and I do not.*

In Bedouin culture, it is not self-evident that “strange” women share personal and sensitive information with each other. However, the mediation creates an opportunity for an intimate encounter between a mediators and pregnant women and mothers. The public space in the MCH clinics provides women an opportunity to share common experiences around pregnancy and motherhood and to create a feminine and supportive “peer group” that crosses tribal and family boundaries. The principle of confidentiality to which the mediators are committed enables them to create trust and connections between women. The mediators indicated the importance and powerful impact of such encounters with women. Nonetheless, exposure to the harsh situations experienced by women overshadows the excitement that arises from the breaking down of barriers and personal and secret sharing:

**S.:** *There are women who want to talk to me about other issues, after the instruction ends. They feel comfortable talking with a person who is not from their family, and they unload everything on the table next to me. Most of the time this is fine. They say what they have to say and unload their burden from their heart and their back and that is it. And they do not want anyone else to know what they told me. This shows how important my role is. It draws the women closer to each other and they become friends here. They get knowledge that they can pass on. I say that even girls who are about to be married, 18 years old, can take this, and tell their sisters and neighbors. Because perhaps they have not heard this. This gives them the urge to do something with this information and therefore they want to pass it on and share it because it was beneficial for them and helped them.*

S. indicates the opportunity created in talks between the mediators and the women coming to the clinics to process and communicate messages and ideas that can “benefit them”. S. was careful to refrain from defining the messages communicated by the medical hegemony as being subversive to the Bedouin faith and tradition and defined them as “beneficial for the women”. She refers to women who come to the clinic as a peer group that can help disseminate medical knowledge and later utilize it when making their own decisions. Medical knowledge provides the mediators pride, strength and motivation to continue their activity.

### Medical knowledge as a source for feminine empowerment

Knowledge and awareness are indicated by the mediators as a mean for achieving control in their lives in general, and particularly in reproductive processes. Veteran mediators who worked in the project for years described their observations of changes that have taken place over time in the women’s responses to the instructions and treatment at the clinics. They described processes of feminine empowerment that are consolidated over the years. The changes in the approach of the patients to the instructions, and their increasing interest in the discussed issues, indicate the growing importance and thirst for medical knowledge as a mean for achieving control and autonomy. S. testified that over the years, she developed a deeper understanding of the significance of each prenatal test, the significance of the test results, as well as possible implications of the test results for their continued lives and the lives of their families. The processes of the growth of awareness to women’s rights to receive healthcare are perceived as a source of power and have broad expressions:

**S.:** *This work is very important. When I began eight years ago women did not have such knowledge. Today they also read and look on the internet and examine and accept my instructions better than at the beginning. The work is very interesting, and I love it.*

**N:** *First all the knowledge they receive, [they] know what it is, and what their rights are and what tests they need to do. They also refer to the nurses more. When I began work, they would not knock on the door or say that they are waiting a long time or ask whether to do this or that test. Today they have more knowledge and are stronger. This is a result of our instruction and [it] indicates the importance of the instruction. I have a responsibility toward the women. I give women awareness and I think that I strengthen them. Knowledge is like giving a person a weapon so he can protect himself. Knowledge is empowerment.*

N. and S. described women’s increasing awareness to perinatal medical knowledge of patients in order to understand and demand answers to health problems. They described empowered women who of their own initiative turn to nurses, and conduct direct talks with professionals, as a good change. This is a dramatic change compared to the past, which is still not enjoyed by all Bedouin women. However, those who did change serve as an example and act as role models for others. Eventually, they quietly undermine the social expectations for passive and submissive behavior expected from women by the hegemony and patriarchal authority.

### Development of Women’s awareness to their pregnant body

Innovative diagnosis and treatment technologies for congenital malformations creates ways of escape for families that are carriers of severe genetic diseases. In this, medicine conveys very significant tidings for Bedouin society. However, the offered medical solutions involve difficult decisions that have social and ethical implications and may undermine traditional beliefs. The mediators are required to communicate medical information and messages that may be interpreted as subversive. Examples of such subversive messages are the implicit (and sometimes explicit) encouragement of marriage outside the tribal group, use of birth control to space between births that also results in a reduction in the number of children, and performing abortions when there is a severe medical risk for a congenital malformation in the fetus. Such messages force the pregnant women as well as the mediators to make very difficult decisions, as they implement deeper understanding of the implications of prenatal diagnosis and preventive perinatal care.

It thus turns out that knowledge is not only an empowering and liberating force, but also a source for anxieties, stress and conflicts, especially in a sensitive and vulnerable situation such as pregnancy. The solutions for reducing the occurrence of congenital defects that medicine can offer the Bedouin community provoke significant resistance, pertaining to both religious and traditional morality and faith. According to the permissive religious Islamic law, it is forbidden to kill an embryo after the first 120 days of pregnancy. Many Bedouin women therefore forego prenatal tests or avoid performance of a termination of pregnancy even after a congenital defect is diagnosed [45, 46].

The instructions given by the mediators stress two hidden messages, which are problematic for the Bedouin community: (1) a recommendation for avoidance of consanguineous marriages; (2) encouragement to perform prenatal diagnosis of congenital defects and considering the cessation of compromised pregnancies due to the high prevalence of consanguineous marriages and consequent hereditary diseases. The following quotes illustrate the intensity of the dilemmas experienced by the mediators and ways in which they cope with the complex task expected of them by the medical system in conveying perinatal knowledge:

**N.:** *I am speaking here of myself. If I am told to assume that I have a bad embryo, and that I need to think [about whether to have an abortion], then I will think twice. If in the end I will kill [the fetus], why? I do not want to. Each one as she sees things. I am not ready. If he is ill, let him come. And if he lives, then maybe God chose that he would live. If not, then he will die, and it was not me who was the cause that killed him, and this is the point. Many of the women also think so. Who am I to cause the baby to die? Who am I to stop this life? I will not tell a woman [what] to choose, I will not say anything. Let us say that she has a foetus with a problem. She needs to do all the tests and there are: 1, 2, 3 possibilities. She needs to choose. I place her at a crossroads of making a harsh decision. In this crossroad it is very difficult to think about complex things, and to stop a life is not easy. I saw women who had an abortion in the fifth month, but I do not know whether [she was] at peace with her decision. For me, as a mother, this is very difficult. Who am I to stop a life? We need to stop the problem [of congenital defects] first, by non-consanguineous marriages. When I speak to parents who want to marry relatives, I tell them let’s do a genetic test and see whether they are carriers [of a mutation for a genetic disease]. I let them think differently.*

N. was the only mediator who agreed to directly and openly confront the dilemma faced by the women. Other mediators only referred to this issue implicitly or chose to circumvent this difficult dilemma. N. referred to this issue from a personal perspective and then from a perspective of the instructed women. Her courageous words do not encompass all dilemmas. Rather, they concentrate on the moral difficulty of terminating a pregnancy, while ignoring other difficulties. For example, the difficulty and suffering involved in the life of a baby born with a congenital defect. The difficulty of mothers to raise children with compromised health is enormous, especially for already disadvantaged mothers. From another angle, for Bedouin women, there is a clear social disadvantage resulting from undergoing genetic tests prior to marriage. Most of the mediators avoided exposing their opinion on these matters during the interview and preferred to use placating rhetoric that does not conflict with the authoritarian medical messages. R. for example preferred to adhere to messages that call on parents to comply with medical recommendations and take responsibility to understand the consequences by themselves:

**R.:** *People should know how much they are responsible for themselves and determine, whether it is by nutrition, by tests, and how much they influence the child’s life. It is true that God created him, but the responsibility for the baby is theirs. For me, the important message is that they will know that there is something that can be done for their child, [and] that they have a responsibility. There are women who have all the test results, and there is something not good in the pregnancy, but they do not want to terminate it. And you can do nothing, and you cannot help her at this point. I wanted them to understand the severity of their problem and not say: Well, whatever comes is welcome. It bothers me that they oppose [the medical recommendations]. But I can also not place the woman into this bubble and force her to do an amniocentesis. I don’t know. It is true that it is dangerous, not too much, but if something would happen to this woman [due to the test], it is not good that the responsibility will be mine. I am afraid to take this responsibility.*

The interviewed mediators explain how they cope with conflictual prenatal situations by sharing their understanding and their own experiences in such harsh decisions from other women’s decisions:

**I.:** *Sometimes I meet with women in the middle of the instructions and they are confused. What to do? Is this good or bad for the baby? Does it do the baby harm or good? [They ask me]: What do you recommend that I do? They ask me things they cannot ask the nurse, because they trust me and feel comfortable asking me. I can tell you that in my first pregnancy, I felt calm even though the first fetal protein test was abnormal. I asked nyself: What should I do? I knew that there was a chance that the results [of the first test] will not be accurate, that they do not necessarily express a problem with the fetus. And I say this also to women, that I had such and such a result … Do all the tests and then make your own decision whether for good or bad.*

In her first pregnancy, I. chose not to comply with all the medical recommendations that she was instructed to communicate as a mediator. She testified that she calmly decided to forego additional tests, knowing that there are some chances for false positive results. Fortunately, despite the indication for an increased risk for a congenital defect, the baby was born healthy and normal. The understanding that this is a screening test and not a diagnostic test put I. at ease. She herself did not comply with the medical recommendation of a more dangerous and invasive diagnostic test that would provide her a certain answer. However, she acted contrary to the recommendation of the medical team, taking a risk and continued with the undiagnosed pregnancy. Sharing her tactic with other women in the clinic raises a problematic message in terms of the conceptions of health promotion and preventive care.

### Social criticism and showing compassion for the mothers of abnormal children

Encounters with women who gave birth to children with congenital defects expose the mediators to dual grievances by their community. On the one hand, religious faith conveys a social expectation that women will continue with their pregnancy even when the fetuses are diagnosed as with compromised health, rarely advising pregnancy termination. On the other hand, the society exhibits very little empathy for children with congenital defects, and often excludes and isolates mothers who gave birth to such babies. H. tells of her acquaintance with a woman whose daughter has Down syndrome. H. described how this acquaintance at the MCH clinic led her to try hard to educate and promote social change in the community’s attitudes toward such women and their abnormal children:

**H.:** *There are those, women who look at such a child [with Down syndrome] with [a very odd expression], and I do not. I regard the child as if she were mine, I hug her and tell her how beautiful she is and how nicely she is dressed. And she [the mother] does not feel that she has a sick child. [This activity] is something in that you can help people who have problems. Besides the tests, you must do things that also are of a great help. I am like the woman’s psychologist. I sit with the women, make them laugh, give them the feeling that I am their friend. If there is a family where I know there is a problem or an illness, I give the feeling that my heart is with them. I do not ask much. I have a neighbor who has a mongoloid son. I talked to her and hugged her and said that this is something usual, any woman can have such a child, and that it is not shameful. When I see her in the street or at a wedding or somewhere, I hug the child and say, wow, what a clean child, what a beautiful child. And then the woman does not feel that she has a sick child.*

H. expresses a nsubtle social criticism of the social alienating responses directed toward mothers of children with birth defects. In this she presents a critical algorithm: the same Bedouin hegemony that advices not to abort fetuses with compromised health should provide the proper support to them and their caretakers-mothers. In her narrative, H. subtly raises a poignant moral question: If we do not encourage early prenatal diagnoses and forbid the termination of pregnancy, why do we not behave with proper compassion for these children and their parents? H. does not act to justify and strengthen the medical message that encourages women to perform early diagnosis and pregnancy termination. Instead, she promotes more moral acceptance for babies and their parents. She criticizes the excluding social attitude by displaying her own compassionate attitude outside the clinic in different public situations and opportunities.

### When the woman is strengthened the community is also strengthened

The interviews with the mediators reveal a pivotal interest in the women’s health and wellbeing. However, most mediators avoided open criticism of the fact that even though women comprise such a vital and important social component in their community, the patriarchal structure ignores many of their rights and needs and weakens them. Coherent and from a feminist perspective, N. well described the position of women in Bedouin society and hinted at the need to refer women’s health and wellbeing issues toward the men:

**N.:** *We are human beings, and this is something that should be looked at with sensitivity. A woman is also something else. This is an entire world. When it is said that the woman is half a society, I say that this is not true, she is the great majority of the society, because she gives birth to the children and educates them and is responsible for them. When a woman is allowed to be strong, we help society to become stronger. When we tell the woman that she should care for her health, then she is the first to care for the health of her children and family. That is why I think that the woman is the body that should be helped. In Bedouin society, the woman was always the party that could be dominated, not the men, and we need to turn to them as well.*

N. directs a critical look at her society, pointing to patriarchy as the source for suppression that ultimately weakens the pregnant female body, even though it is a central axis in the conservation of the society. It is not easy for women who were raised in a patriarchic and suppressive environment to express overt criticism and point to the men as the source of suppression. However, N. does this from an empowered position, not as a victim of suppression. In contradistinction, W. told of her personal experience under masculine domination that was non-supportive and even neglectful of basic needs:

**S:** *I know what it is to be a hungry child. Once my husband asked his brother to buy milk for my son. He said OK, at ten I will bring you milk. And this was a five-months old child. How many hours does one have to wait until milk is brought? In the end he brought milk, but only for his children. If I had gone crazy it would only have been for this state of my children. But I slowly built myself, praise Allah. I had a difficult life, and none of my husband’s brothers offered help, and the distance between us is only ten meters. What scares the women is their husbands is their fear from disappointment. Perhaps he will not take me to the clinic, who will take me for tests? The women do not even go for genetic tests.*

As someone with first-hand experience of the degrading dependence on men, S. well understands the difficulties that other women still experience, particularly regarding satisfaction of medical needs for women who are dependent on men chaperons and transportation to and from the clinic. Today, when S. drives her own car and brings home a modest salary from her work as a mediator, she substantially reduced her dependence on her husband and his family. As someone who has regular and direct access to health services, S., like the other mediators, understands that coming to the clinic for routine monitoring of the pregnancy is not something taken for granted by many Bedouin women.

### Seeing the change from inside, and acting from the outside

It should be noted that even today, the mediators need the support of their patriarchal families in order to work as mediators. During the first years of the project they were careful to refrain from any encounter with Bedouin men who came to the clinics as escorts, so as not to endanger their reputation as modest women and consequently the consent they received from their family to continue with their work. Men accompanying their wives to the clinics usually stood outside the clinics, in order to avoid contact with other women. They often did this impatiently in their cars while blowing the horn to the women waiting inside. When the queues were long, the women were sometimes obliged to return to their homes without reaching the reception.

B., one of the veteran mediators, recalls the first days, and indicates a gradual and significant transformation that took place among the men, who began to express an interest in the content of the instructions. Concomitantly the mediators are gaining self-confidence and social support to approach and instruct them.

**B.:** *The change was very big. I saw the women when I first began my work there, and today they are not at all like the women then. Today they are more active, but at the beginning it was very difficult for the women. Everything was difficult. Once the husband would take an hour off from work, would bring the woman to the clinic and would wait for her by the door. This was stressful for all of us. Today, most of the women arrive alone. I give the instruction happily. Not long ago I instructed a woman at the mother-child health clinic. She was escorted by a man and I did not feel comfortable in his presence. I saw that his wife is also stressed by his presence. He was a frightening man. He shouted at the nurse that he is waiting a long time. I said to myself, B., do what you can do. I took a deep breath and asked him to come into my room. I instructed the wife on taking folic acid during the pregnancy while he was outside the door. He came in after her and began to show an interest in my work and asked questions. I explained the importance of taking folic acid during pregnancy to him, and he said that in that case, he will go and buy the vitamin that I recommended for his wife. The nurse said that I succeed in persuading the men and therefore she will refer men to me for instruction. But there are men who do not accept instruction from an unknown woman.*

A different aspect of change is explained by N., who interpreted men and women’s relationships with her critical social eye:

**N.:** *I think that in Bedouin society women are the weakest body. Women were given much information, but not the men, and this is a problem. We strengthen the weak party and the other party dominates her and holds everything in its hands. When we think only of the women, this is something that needs to be changed, and we need to think of the men as well.*

*I feel that I give the women tools that will change their lives and the future of the coming generations. That this generation will not suffer like the previous generation. I am optimistic and love to help and change things, very slowly. After all my experiences, one needs some patience and slowly things will change.*

N.’s assertion that change will be mobilized through the achievement of men’s understanding of the importance of prioritizing women’s needs connects well with B.’s above-mentioned experiences with involving men in the instruction. However, N.’s interpretation of gender relationships is more analytical and theoretical. Another agenda of seeing change through her work as a mediator is expressed by S:

**S.:** *I push the women to continue in their own way … The message to the women should be that they need to be independent and have a goal in life. A person without a goal is nothing. I love women who take charge of their lives. They listen to me and go out of the house. First, the knowledge that they receive, they know their rights and what tests they should do. They also go to the nurses more often. At the beginning they would not knock on the door. But today they comment that they have been waiting a long time or ask when this or that test should be performed. They have more knowledge and are stronger, and this is a result of our instructions. I see the confidence and the spark in their eyes, and it makes me happy that I accomplished something. That I changed something.*

Here S’s words focus on women as change agents rather than on gender relations. The common denominator of all above narratives is that mediators see their work as an opportunity for substantial, even if gradual and slow, social change.

### Promoting Women’s health from “the middle”

The most prominent theme in the interviews with the mediators is their role that positions them “in the middle”. They described themselves as situated in the middle, turning their role of mediation into complex but pivotal role on several levels: between the medical system and the women who visit the clinic; between women who are bound to the values of a traditional society and traditional patriarchy; between their bodies as pregnant women and their fetus, as authentically expressed by I.:

**I.:** *I saw that the mediation is the relation between me and the woman, between the woman and the nurse, with me in the middle. I also saw myself between the [prenatal] tests and the important things for the woman and her fetus and saw myself intervening between the woman and her baby. It is as if I grab the rope wherever it wants to go, help her walk if she cannot walk; help her to get up and to sit down; explain to her where she is found … Most of the time the special position of being in the middle is a comfortable place. [However] I sometimes see myself in the wrong place because I interfere in something personal. I see that the baby and the woman’s tests are something personal between her and her baby and I interfere, and sometimes this is for the better and sometimes for the worse.*

I.’s sayings provide a description two contradictory meanings of “in the middle”: one connecting between medical services and the women and Bedouin society and the other creating a barrier and separation between entities that are naturally and strongly connected. Being “in the middle”, with all its aspects, always raises questions, discussions, and critical self-reflection.

Medicine, in I.'s view, sometimes creates an artificial disconnection between women and their pregnancy, and a gap between women-mediators and their community. Even graver, I. finds herself in a position that may separate between a woman and her fetus, when the medical information she makes accessible may create a separation-termination of pregnancy. The magnitude of the dilemma makes I. think very hard about her role as a health mediator, but these thoughts remain vague. She prefers to focus on the position of a woman who leads “the rope wherever it wants to go” and to help women orientate, stand, walk and reach “their correct place”. In our interpretation of her words, the place to where “the rope leads” remains obscure.

## Discussion

This article explores the ways by which Bedouin women who work as perinatal health mediators conduct complex and complicated negotiation over a renewed definition of their status and the status of their peer group, within a network of power relations and dominance. The main emphasis is on their status of women as carrying the pregnancy and giving birth, caregiving, caring and nourishing. The negotiation is conducted in parallel vis-à-vis two hegemonic entities with power and authority: the men in the patriarchic Bedouin society and the health professionals in the perinatal health services. They are in constant daily contact with the former source of authority and meet the latter during the course of their work and as patients, and during their own experiences with pregnancy and birth, as mothers and as caretakers.

As women who are part of a traditional society in which drastic processes of change are taking place, drawing near to modernity creates an unusual opportunity to cross boundaries and adopt new ideas and points of view, such as a feminist theoretical perspectives on universal gender equality and rights to health. These, according to Herzog [35], change the social agenda via selection processes, conceptualization, labeling and fixation of social categories. Hertzog’s viewpoint of origin is that society is a system of constant struggle over setting and undoing boundaries. It is therefore interesting to look at the institutionalized arrangements, but also at those who were pushed to the margins and those that undermine the existing order, as well as the changing interrelations that exist between all these arrangements within and between the struggle arenas. This observation well reflect the situation of women health-mediators who wish to change their position without defying existing institutions and authorities both in the medical arena or in their own communities as patriarchal and restricting they are. Fundamental changes in gender relations are deeply rooted in the culture regarding power, authority and gender roles, and particularly regarding pregnancy and birth. They also carry a great risk for structural disintegration of the social and family foundations that are guided by belief, religion and tradition. Furthermore, as indicated by several scholars, overt and subversive feminine empowerment may exacerbate patriarchic suppression and make the situation worse for the women. As noted previously by other scholars [3, 19, 25], the women who participated in this research act in sophisticated ways to maintain their social relationships while striving for change.

The role of health promotion, instruction and mediation around the perinatal issue is strongly connected to the feminine essence in traditional society and imparts the mediator’s legitimization to act within a social consensus on issues that are considered legitimate. Preservation of their health as women, with reference to reproduction, is perceived as a mean for increasing feminine commitment to traditional gender roles: giving birth to offspring, caring for their family. In practice, the mediators, and through them other Bedouin women, communicate messages that undermine the control and restrictions levied on them by the men and increase their freedom of movement in the public space for monitoring their pregnancy and the babies’ development. However, this is a mixed blessing. Consistent with previous research on Bedouin women in the interface with perinatal health [63, 64], increasing compliance with recommendations of the health institutions during and after pregnancy may also restrict the autonomy over the pregnant feminine body, this time via medicalization. The mediators witness and share in drawing near to modernity and creating an autonomic feminine prenatal space that leads to increased medical control over the pregnant women’s body.

The women who comply with the medical recommendations often find themselves facing difficult dilemmas that arise when prenatal tests indicate problematic pregnancies [11, 47]. In situations where pregnancy termination is initiated for a medical reason, compliance with Western medicine which apparently helps women create freedom and control leads them to acute conflicts with their moral values and the Muslim-Bedouin belief and tradition. This is also true for questions regarding the continued tradition of consanguineous marriages and support pervious research finding [46, 47, 64, 65]. The mediators find themselves in the middle, due to their role in the health services, where they encourage women to comply with medical recommendations, but understand that by this they cause the women to face a whole new set of dilemmas. Furthermore, the dilemmas do not end there, but rather become even more scathing later in life: whether they need to decide to continue or terminate the pregnancy. As described by the women, they continue to carry the dilemma a long way after the abortion's decision.

The mediators claim that knowledge is power. “A weapon” they can impart to the weakened women-mothers by whose means they can increase control over their lives, their health and the health of their children. However, in practice, dilemmas, concerns, and fears are born together with the knowledge and the power, and the liberation from patriarchy is relative and limited. The mediators, and with them additional women, are not interested in an overt confrontation with hegemonic patriarchic structures. Rather, they desire to redefine the position of pregnant women, mothers, caregivers, who care for and nurture their families. In this sense, the right to health and the introduction of an aspiration to conscious educated and active feminine action for their health and the health of their children carries tidings in the spirit of feminists such as Tronto [32, 33], Gilligan [34], Hankivsky [29], Mann [31]) and others. These feminists search for a way to preserve the feminine affinity to gender skills that characterize women, without losing their status and society’s appreciation of their activity in the reproductive and family field.

In a somewhat different spirit, de Certeau [66] wrote about the quiet way, avoiding overt conflict, of the weakened to redefine their position in the public space. The encounter with the health services affords the mediators legitimization to reshape the rights and even strengthens the commitment of pregnant women to preserve their health and the health of their fetuses. The arena of the clinic enables an encounter of the mediators, Bedouin women belonging to the peer group, and enables transmission of messages, some of which are found at the heart of the Bedouins’ social consensus. Others are subversive and try to undermine traditional structures of family, gender caregiving roles, feminine autonomy over the pregnant body and decisions regarding the future of the pregnancy and the fetus-newborn.

The common ground in the narratives presented by the mediators enables a look into the negotiation over the boundaries of power and autonomy taking place in an arena of perinatal feminine help and care. On the one hand, the Bedouin women use the mediator role for bursting the boundaries of patriarchic domination and for realizing their abilities outside the traditional domestic sphere. On the other hand, they want to preserve the social structure and to hold a dialogue with the men and convince them to come over to their side. They consider that in a total war they will lose, and suppression will increase, and that they will achieve more with slow work that connects the men to the women’s needs and for the strengthening of the entire society.

The negotiation that takes place in this dense network of interrelations does not take place under conditions of equality. On the contrary, this is a forceful encounter (even if there is no overt and blatant collision) in which a constant struggle takes place between preservation and change. The women struggle for the definition of their identity and boundaries, in parallel to the nature and essence of society. The mediators’ avoidance of overt collision with patriarchy, and the attempt to draw the men to a discourse of promoting the pregnant women’s health, best illustrate the desire to generate change and strengthen their status without erosion of existing social structures. Such a course requires not only caution in order to protect themselves against a harsh reaction, but also awareness, strength, restraint, and most of all patience for processes that take a long time.

## Conclusion

The feminist approach that aspired to draw women from weakened minority groups to the West out of an elitist sense that the solution to all suppressions lies in the West requires a second thought, as its price was paid by women who became “victims of modernization” [43]. The narrative presented by the health mediators about one decade after the above criticism is not a victimized narrative of a beaten group (although many paid prices on the way), but rather a narrative that strengthens gradually and in measured steps. It includes drawing toward the West but does not even hint at rejecting or undermining Bedouin tradition and culture.

In this sense, it is possible that these mediators personify a breakthrough in feminist thinking. If this is indeed so, then the new feminist metamorphosis has ceased dealing in romanticizing modernity and depreciating tradition. It is apparent that the mediators adhere to their ethnic and cultural identity and view modern medicine as a mean for improvement and not as an ax to grind against culture. Perhaps the most powerful expression for this is their internal ability to criticize and defy the exclusion and lack of compassion for women who give birth to babies with congenital defects. Only a person whose culture is important to him will defy the injustice and internal inequality and will aspire to improve and sophisticate patterns that should be denounced. Their criticism is voiced out of love for the society from which they come and which they strive to improve.

The medical messages expose the women, and with them the mediators, to distress and to a crossroads where they are required to look at a problematic pregnancy and make difficult and complex decisions: between tradition and religion and medicine; between the community and the women and their body; between the women and their fetuses. The mediators themselves are shaken by the intensity of the dilemmas to which they are exposed as mothers, as relatives of affected people, as women whose job is to present medical messages with which they do not completely agree. Their burden is sometimes intolerable, but the opportunity they have as agents of change and personal empowerment in their peer group is too valuable to drop.

### Limitations of the study

Narrative analyses are not intended to determine a homogeneous position, state of mind, or similar reaction toward social situations and challenges in life. Rather, they are directed toward gaining an understanding of the common ground that exists in the accumulated diverse narratives and finding the common axis that will make sense and be relevant to complex social situations within the dynamics of social processes taking place in global as well as local contexts. We therefore limit our analysis to the agents who participated in a specific point in time but aspire to provide a wider perspective.

### Supplementary Information

**Additional file 1.** Interview guide (translated to English).

## Data Availability

Interviews as well as data documentation during the project were conducted, transcribed and analyzed in Hebrew. We will be able to provide recorded raw data material upon request, without personal details for the preservation of the interviewees’ confidentiality.

## Appendix

**Table 1:** Characteristics of the informants (women mediators) at the time of the interviews

Name’s initial letter	Age	Marital status + children	Number of attending clinics (total working days in a week)	Location of clinic	Seniority in the project
M.	30–40	Married + 2–4	2 (5)	Planned Bedouin town	6–9 years ^a^
L.	30–40	Married + 2–4	1 (3)	Bedouin village	3–5 years ^a^
A.	~ 30	Married + 5–7	1 (2)	Bedouin Village in process of recognition	1–2 years
S.	~ 35	Married + 2–4	2 (4)	Town serving the Bedouin population	6–9 years ^a^
R.	20–30	Single/divorced	2 (4)	Bedouin village	3–5 years
B.	~ 30	Married + 5–7	2 (5)	Planned Bedouin town	Veteran, 10 years and more
H.	40–50	Married + 8–10, grandmother	3 (5)	Planned Bedouin town	Veteran, 10 years and more
I.	20–25	Married + 2	1 (5)	Bedouin village	3–5 years
F.	30–40	Married + 3	1 (4)	Bedouin village	6–9 years
S.	40–50	Married + 8–10 grandmother	1 (3)	Bedouin village	6–9 years ^a^
N.	40–50	Married + 1	2 (5)	city serving the Bedouin population	3–5 years

^a^Not consecutive

## Abbreviations

FOH: Faculty of Health Sciences
MCH: Mother-and-child clinics
MOH: Ministry of Health
