# Supplementary material for: Between a woman and her fetus: Bedouin women mediators advance the health of pregnant women and babies in their society
Source: BMC Pregnancy Childbirth. 2021 Mar 6;21:190. doi: 10.1186/s12884-021-03661-4 (PMC7937305; doi:10.1186/s12884-021-03661-4)
Supplement: Supplementary file 1 — Additional file 1. Interview guide (translated to English). [file 12884_2021_3661_MOESM1_ESM.docx]

Additional file 1:

Interview guide (translated to English):

Please present yourself, your family, your education, marriage, and your place of residence

Please tell me about your work as health mediator in this project:

When did you started?

What kinds of training did you get? What was the most meaningful parts of these trainings for you? And why?

Where is the location of your working place/s? What does it take for you to reach your workplace? (transportation, walking, traditional barriers, etc)

How long do you work there?

Tell me more about your experience as a mediator:

Challenges, difficulties, benefits, etc.

Can you tell me about conflicts you face in your work?

What are the most interesting and meaningful parts of the instructional materials you convey to the public for you and why so?

Are there parts of the instructional materials you are not comfortable with? And if so, why and what do you do with that?

Tell me about the atmosphere in the MCH clinic. Your relationships with the personnel,

your relationships with the women who come to the (MCH) clinic.

Tell me about comments and responses you hear from members of your community (women, men/husbands, family members, others)

What did you learn from your work about your community?

How have your perspectives changed or developed over the years of your work?

What skills have you developed over time?

What do you think should be changed/conserved in this project?

What is the importance / unimportance of this project from your point of view?

Do you have any other comment that you want to share with me?

Thank you for sharing your views with me.
